# Supplementary material for: Giant room-temperature nonlinearities in a monolayer Janus topological semiconductor
Source: Nat Commun. 2023 Aug 16;14:4953. doi: 10.1038/s41467-023-40373-z (PMC10432555; doi:10.1038/s41467-023-40373-z)
Supplement: Supplementary file 1 — Supplementary Information [file 41467_2023_40373_MOESM1_ESM.pdf]

Supplementary Information

Giant room-temperature nonlinearities in a monolayer Janus topological semiconductor

Jiaojian Shi[1,2,†], Haowei Xu[3,†],Christian Heide[4,5], Changan HuangFu[6], Chenyi Xia[1,2], Felipe de Quesada[1,2], Hongzhi Shen[7], Tianyi Zhang[8], Leo Yu[9], Amalya Johnson[1], Fang Liu[5,10], Enzheng Shi[7], Liying Jiao[6], Tony Heinz[5,9], Shambhu Ghimire[5], Ju Li[3,11], Jing Kong[8], Yunfan Guo[12*], Aaron M. Lindenberg[1,2,5*]

[1]Department of Materials Science and Engineering, Stanford University, Stanford, California 94305, United States

[2]Stanford Institute for Materials and Energy Sciences, SLAC National Accelerator Laboratory, Menlo Park, California 94025, United States

[3]Department of Nuclear Science and Engineering, Massachusetts Institute of Technology, Cambridge, Massachusetts 02139, United States

[4]Department of Applied Physics, Stanford University, Stanford, California 94305, United States

[5]Stanford PULSE Institute, SLAC National Accelerator Laboratory, Menlo Park, California 94025, United States

[6]Key Laboratory of Organic Optoelectronics and Molecular Engineering of the Ministry of Education, Department of Chemistry, Tsinghua University, Beijing 100083, China

[7]School of Engineering, Westlake University, Hangzhou, 310024, China

[8]Department of Electrical Engineering and Computer Science, Massachusetts Institute of Technology, Cambridge, Massachusetts 02139, United States

[9]E. L. Ginzton Laboratory, Stanford University, Stanford, California 94305, United States

[10]Department of Chemistry, Stanford University, Stanford, California 94305, United States

[11]Department of Materials Science and Engineering, Massachusetts Institute of Technology, Cambridge, Massachusetts 02139, United States

[12]Key Laboratory of Excited-State Materials of Zhejiang Province, Department of Chemistry, State Key Laboratory of Silicon and Advanced Semiconductor Materials, Zhejiang University, Hangzhou 310058, China

*Corresponding author. E-mail: yunfanguo@zju.edu.cn (Y. G.); aaronl@stanford.edu (A. L.)

†These authors contributed equally to this work.

Supplementary Note 1. Estimation of photo-responsivity and noise-equivalent power of THz detectors based on 1T' JTMD

The photo-responsivity of the THz detector is defined as the DC photo-current I_{DC} divided by the absorbed THz power P_{abs}

$$\mathcal{r} = \frac{I_{\mathrm{DC}}}{P_{\mathrm{abs}}} \qquad \text{(Supplementary Equation 1)}$$

Using the geometry shown in Fig. S11, the DC current can be expressed as

$$I_{\mathrm{DC}} = \frac{1}{2}\xi(\omega)E^2wd \qquad \text{(Supplementary Equation 2)}$$

where E is the electric field strength of the incident THz radiation. $\xi(\omega)$ is the second-order photoconductivity (e.g., the shift current conductivity). From our ab initio calculations, $\xi(\omega)d$ of 1T' JTMD is on the order of 10^3 nm · μA/V^2 in the THz range[1]. Meanwhile, the absorbed power of the 2D flake is[2]

$$P_{\mathrm{abs}} = P_0\left[1 - \exp\left(-\frac{\sigma_r(\omega)d}{c_0\varepsilon_0}\right)\right] \approx P_0\frac{\sigma_r(\omega)d}{c_0\varepsilon_0} \qquad \text{(Supplementary Equation 3)}$$

Here $P_0 = \frac{1}{2}c_0\varepsilon_0E^2wl$ is the incident power, c_0 is the speed of light, and ε_0 is the vacuum permittivity. $\sigma_r(\omega)$ is the real part of the first-order conductivity, and one has $\sigma_r(\omega)d \sim 5 \times 10^4\ \mathrm{nm} \cdot \mathrm{S/m}$ in the THz range, according to our ab initio calculations. Then, one has the photo-responsivity of the 1T' JTMD THz detector as

$$\mathcal{r}(\omega) = \frac{I_{\mathrm{DC}}}{P_{\mathrm{abs}}} = \frac{\xi(\omega)}{\sigma_r(\omega)l} \sim 0.2\ \mathrm{A/W} \qquad \text{(Supplementary Equation 4)}$$

as shown in Fig. 4 in the main text. Here we used[3,4] $l = 0.1$ μm.

Next, we estimate the noise-equivalent power (NEP) of the 1T' JTMD THz detector, which can be obtained from

$$\mathrm{NEP} = \frac{\sqrt{4k_BT/R_0}}{\mathcal{r}}, \qquad \text{(Supplementary Equation 5)}$$

where k_B is the Boltzmann constant, T is the temperature. The resistance of the JTMD sample can be estimated with

$$R_0 = \frac{1}{\sigma_r(\omega = 0)}\frac{l}{wd} \approx 2 \times 10^4\ \Omega, \qquad \text{(Supplementary Equation 6)}$$

which is comparable with the experimental results[4] of 1T' MoS_2. Here we used $l = w$. Finally, one has

$$\mathrm{NEP} \sim 5\ \mathrm{pW}/\sqrt{\mathrm{Hz}}$$

in the THz range (Fig. 4 in the main text).

Supplementary Note 2. Additional information about HHG efficiency comparison with other samples

For comparison with 17th-order HHG at ZnO, our data show similar cutoff orders and signal-to-noise ratios as the ZnO data[5] under similar incident MIR fields at ~ 0.2 V/Å despite on a monolayer flake (< 1 nm) with ~10 µm lateral sizes. The 500-µm ZnO used is theoretically shown to have an effective thickness of 100-200 nm[6], meaning the HHG efficiency per thickness at 1*T'* MoSSe is at least 100 times higher. When taking the lateral size (~10 µm) and beam spot size (~100 µm) into account, the 1*T'* MoSSe HHG could be 1000 times higher than ZnO. For 18th-order HHG, ZnO is centrosymmetric and does not generate even-order harmonics. Similar comparisons are performed for other solid samples in Table 1.

Supplementary Note 3. Estimation of momentum change in intraband electron acceleration

We can estimate the distance using the semi-classical equation of motion of the electrons: $\frac{\partial k}{\partial t} = \frac{eE}{\hbar}$, where E is the electric field. This equation yields $\Delta k \approx \frac{\tau eE}{\hbar}$, where τ is the decoherence time of the electrons. Taking $\tau = 1$ fs and $E = 1\ \mathrm{V/nm}$, one has $\Delta k \approx 0.15\ \text{Å}^{-1}$, which is 1 ~ 10 % of the size of the Brillouin zone.

Supplementary Note 4. Sample stability discussion under thermal and laser excitation

From the energetic perspective, the 2H phases should be more stable than the 1T' phases, as reported in Fig. S7 in the Supplementary of Ref. [7] The 1T' phase is a metastable state, and its energy per unit cell is around 0.5 eV higher than that of the 2H phase. From the practical perspective, the Janus structure is less stable than the non-Janus structure thermodynamically. For its stability under MIR excitation, we did not observe any evident sample damage in either 1T' or 2H phase MoSSe under the highest possible MIR fluence available in our lab.

Supplementary Note 5. Additional discussion on the angle and fluence dependence in TES measurements

The sinusoidal angle dependence with a period of π shown in Fig. 3c in the main text is a typical feature of second-order nonlinear optical responses. This is because the total response is $R \sim E \cdot \chi^{(2)} \cdot E$, where $\chi^{(2)}$ is a rank-2 tensor. If the polarization of E is rotated in the x-y plane with an angle of θ, then one has $R(\theta) = E^2\left[\chi_{xx}^{(2)} \cos^2\theta + \chi_{yy}^{(2)} \sin^2\theta + 2\chi_{xy}^{(2)} \sin\theta\cos\theta\right]$. Hence, the sinusoidal angle dependence appears as long as $\chi^{(2)}$ is not proportional to identity tensor. From

such angle dependence, one can assess e.g., if there is a mirror symmetry. But usually, one cannot get full information on the crystal symmetry (e.g., space group) from these angle dependences. Similar angle dependence data and microscopic origin above has been reported in Ref. [8]. For the fluence dependence data shown in Fig. 3d, the signal continues to increase at higher fluences, albeit with a smaller gradient. Such phenomena are likely due to two combined effects: (1) Saturation of photocurrents due to carrier generation, as reported in Ref. [8]; (2) Nonlinear photocurrents that leads to increasing THz emission signal as a function of excitation fields.

Supplementary Note 6. Additional discussion on TES and SHG measurements in 2H-MoSSe and 2H-MoS_2

Regarding 2H-MoSSe and 2H-MoS_2, they show vanishing TES signal and detectable SHG signal, which is likely due to a resonant effect. SHG concerns the sum-frequency process, which is characterized by $\chi^{(2)}(\omega,\omega;2\omega)$, where ω is the input frequency. For 2H MoS_2 and MoSSe, the doubled frequency 2ω (around 3 eV) is above the bandgap (below 2 eV). Consequently, the SHG can be resonantly boosted. In contrast, the TES process is characterized by $\chi^{(2)}(\omega_1,\omega_2;\omega_1-\omega_2)$, where ω_1 and ω_2 are two input frequencies, while $\omega_1-\omega_2$ is the difference frequency and is in the THz range. In our experiments, ω_1,ω_2 and $\omega_1-\omega_2$ are all below the bandgap of 2H MoS_2 and MoSSe. Hence, the TES for the 2H-phase structures is basically a non-resonant process and is intrinsically weaker than the resonant SHG. In addition, the signal levels in these two independent experiments are also influenced by the different detection sensitivities for TES and SHG.

Supplementary Note 7. Additional discussion on incident angle dependence in SHG

The angle dependence is dominated by the crystal structure. For example, if the crystal structure is isotropic with e.g., octahedral symmetry, then there should be no angle dependence. On the other hand, if the symmetry of the crystal structure is lower, then the angle dependence can appear, because electric fields with different polarization "feels" different atomic environment, and the optical responses would be different. On the other hand, topological properties typically do not have a significant impact on angle dependence. For instance, isotropic structures, regardless of whether they possess topological properties or not, should not display any angle dependence.

Supplementary Note 8. Additional discussion on nonlinear susceptibility calculation

Note that the SHG of 2H MoSSe in Fig. 4e seems small because that of 1T' MoSSe is huge at small frequencies. In other words, the scale of the y-axis in Fig. 4 is large. The nonlinear susceptibility $\chi^{(2)}$ of the SHG of 2H MoSSe is on the order of $1\ \mathrm{nm/V}$, which is quite large (see also Supplementary Fig. 14). The SHG $\chi^{(2)}$ of typical crystals is on the order of $10^{-3}\ \mathrm{nm/V}$. We also note that the specific tensor elements shown in Fig. 4 are chosen because they are relatively large among all tensor elements. The other in-plane elements of the SHG tensor are shown in Supplementary Fig. 14.

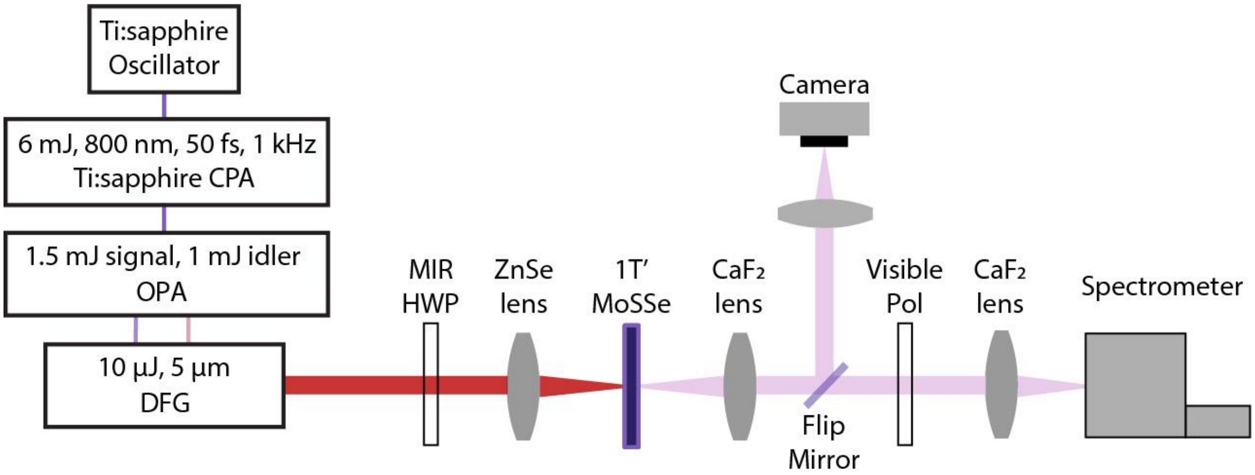

Supplementary Fig. 1 | Schematic illustration of the HHG setup. An amplified Ti:sapphire laser system is used to pump an optical parametric amplifier (OPA, TOPAS-HE, Light Conversion Inc.). The signal (~ 1300 nm) and idler (~ 1900 nm) from the OPA are mixed in a GaSe crystal (Eksma Optics Inc., *z*-cut, 0.5 mm thick) for difference-frequency generation. The resulting mid-infrared radiation is cleaned by a bandpass filter, centered at 5.0 μm (Thorlabs Inc., FB5000-500). The polarization of the MIR beam is controlled by a zero-order MgF_2 half-wave plate (HWP) and focuses on the sample with a 15 cm focal length lens. The spot sizes are 100 μm (1/e^2 intensity radius) for the MIR beam. A 20× Mitutoyo microscope objective attached to a CCD camera can be placed directly behind the sample to characterize the spot size at the focal plane and align the spot on the sample. The spot size of the MIR-beam is characterized with a beam profiler (Dataray, WinCamD-IR-BB). The generated high harmonics are collected and focused by CaF_2 lenses and directed into a spectrometer equipped with a thermoelectronically cooled CCD camera (Princeton Instruments Inc., Pixis 400B). All HHG spectra were measured in a transmission geometry and under ambient conditions.

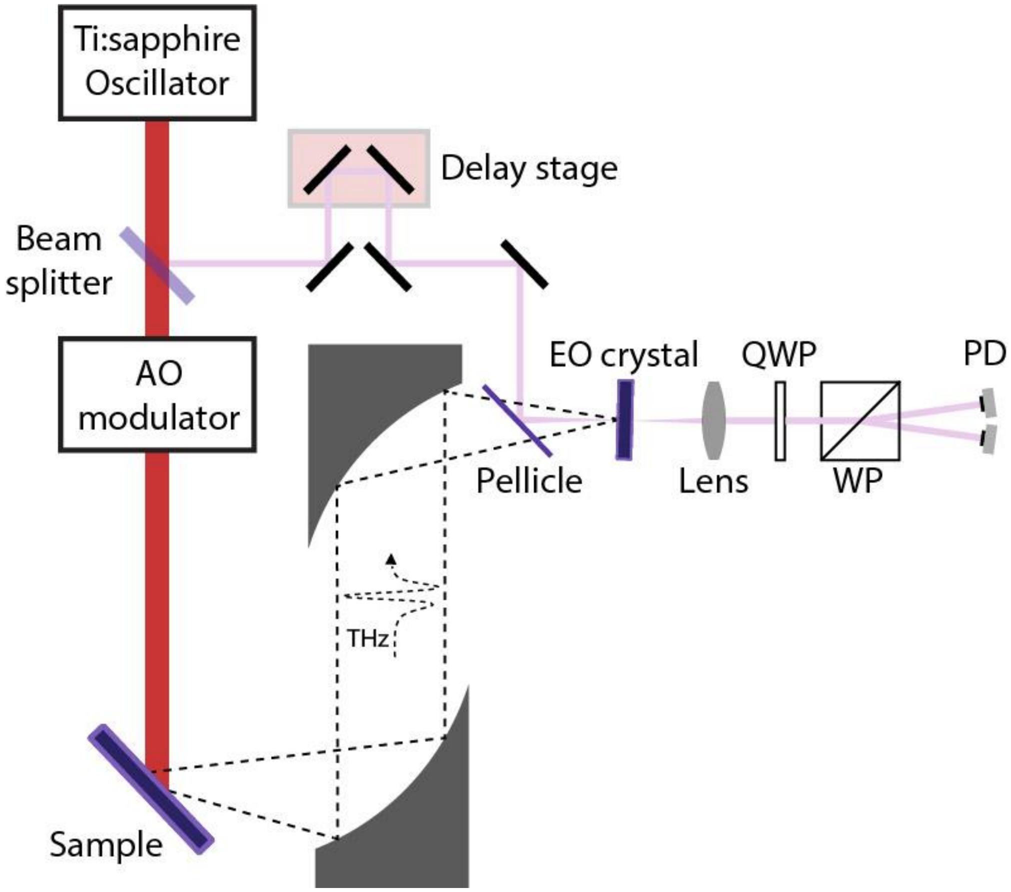

Supplementary Fig. 2 | Schematic illustration of the TES setup. A long-cavity Ti:sapphire oscillator (5.12 MHz repetition rate) is used to pump the sample for THz generation. The residual 800-nm beam after the sample is a 500-µm-thick high-resistivity Si wafer. The pump beam is modulated by an acousto-optic modulator for lock-in detection. The generated THz emission is focused on a electro-optic (EO) crystal, which is either a 1-mm thick ZnTe or a 258-µm-thick GaP. AO: acousto-optic, WP: Wollaston prism, PD: photodetector, QWP: quarter-wave plate.

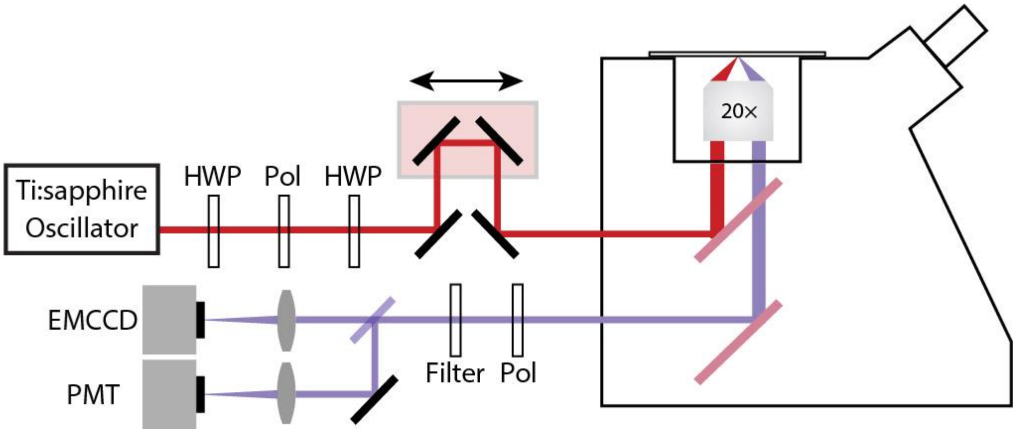

Supplementary Fig. 3 | Schematic illustration of the angle-resolved SHG setup. The fundamental beam is provided by a Ti:sapphire oscillator and coupled to a 20× objective for SHG measurements. The beam can be displaced perpendicular to the beam perpendicularly to the beam direction by moving the translational stage, which enables angle-resolved SHG measurements. The collected SHG signal is detected by either an electron-multiplying charge-coupled device or a photomultiplier tube.

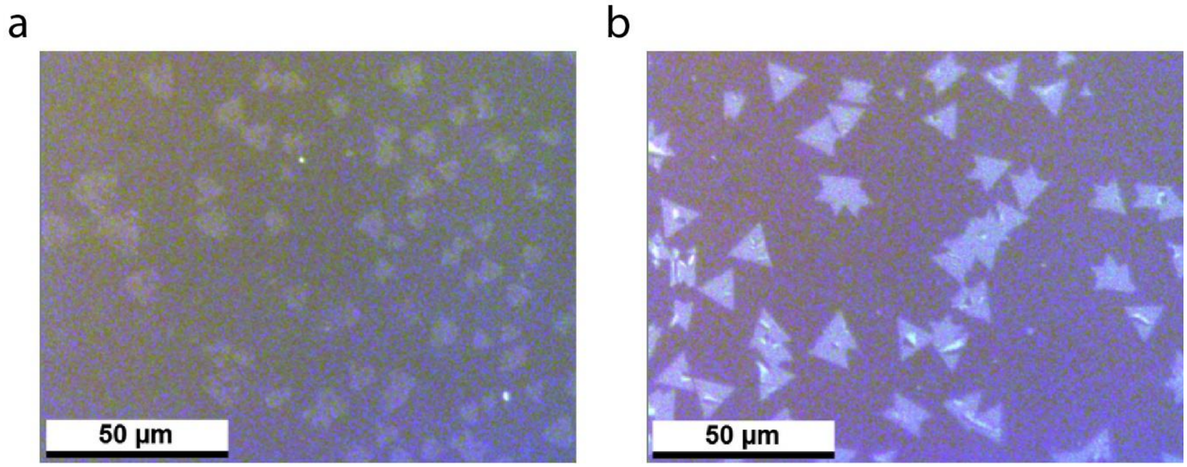

Supplementary Fig. 4 | Optical images of 1*T′* MoSSe (left) and 2*H* MoSSe (right). The spacing between the flakes are randomly distributed. Experimentally, we irradiated a single flake in a sparsely populated area and confirmed that we did not experience any interference from neighboring flakes. We achieved this by measuring the polarization dependence and observing the near-perfect cancellation of HHG at specific angles to one of the crystallographic axes.

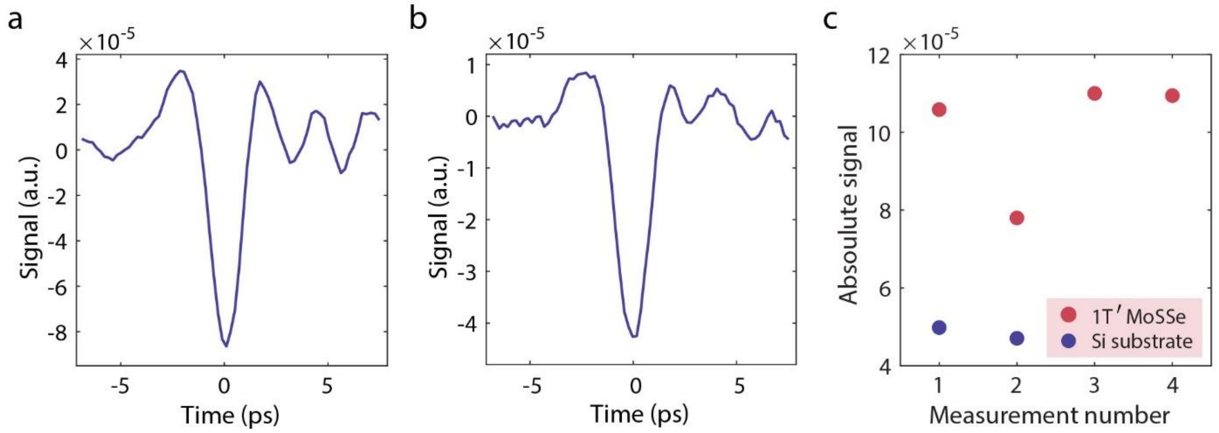

Supplementary Fig. 5 | TES measurements on 1*T*′ MoSSe grown on silicon and silicon substrate. **a**, THz emission signal from 1*T*′ MoSSe grown on silicon. It contains both signals from 1*T*′ MoSSe and the silicon substrate. **b**, THz emission signal from the bare silicon substrate. **c**, Measurements on different sample spots show that THz emission intensities in 1*T*′ MoSSe sample are about two times from the bare substrate, meaning the signal from 1*T*′ MoSSe is comparable to those from the silicon substrate.

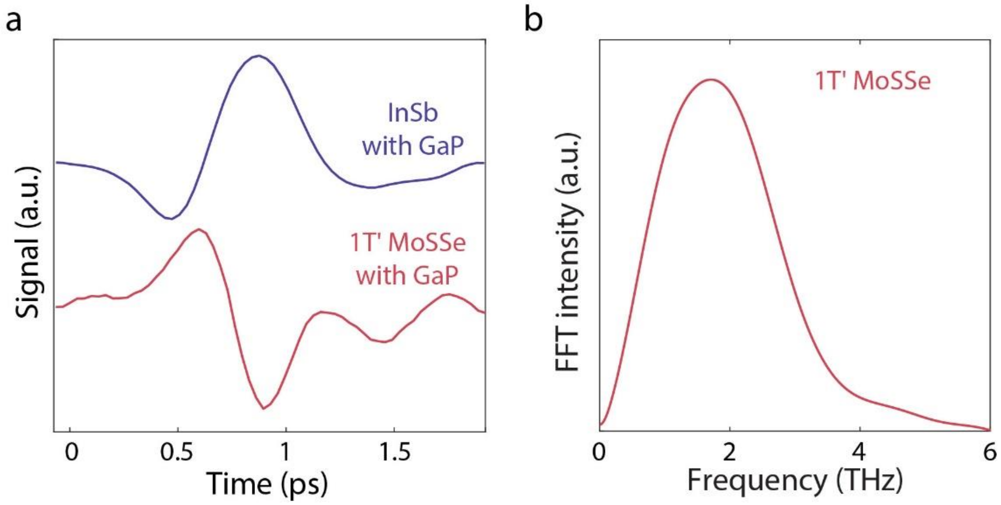

Supplementary Fig. 6 | TES experiments on InSb and 1*T′* MoSSe measured with gallium phosphite crystal. a, TES measurement on reference sample InSb and 1*T′* MoSSe. The EO crystal here is GaP crystal that can measure higher frequency components than the ZnTe crystal used to obtain results reported in the main text. **b**, THz emission spectrum of 1*T′* MoSSe shows a center frequency at about 1.6 THz and extends up to 4 THz.

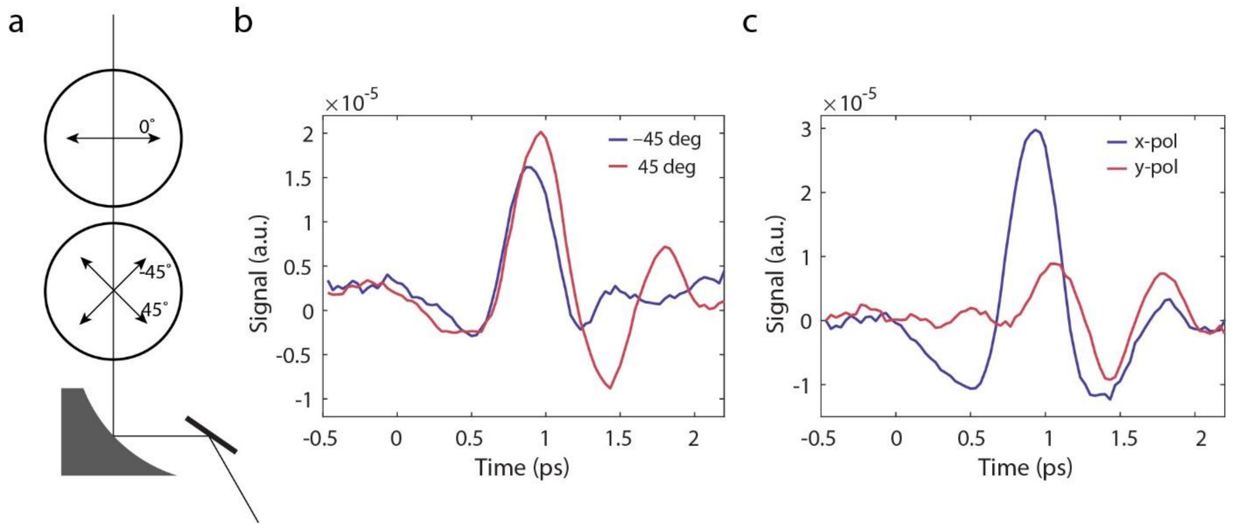

Supplementary Fig. 7 | THz emission polarization analysis with linearly polarized 800-nm pump. **a**, schematic illustration of THz emission polarization analysis. The polarization of the second polarizer (along the beam) was fixed to be horizontal (0 degree). The polarization of the first polarizer was chosen to be 45 degree or -45 degree for comparison. **b**, THz emission traces at 45 degree and -45 degree polarization angles. **c**, THz emission traces with *x* and *y* polarization inferred from **b**, which shows the emission is mainly polarized along the *x*-direction.

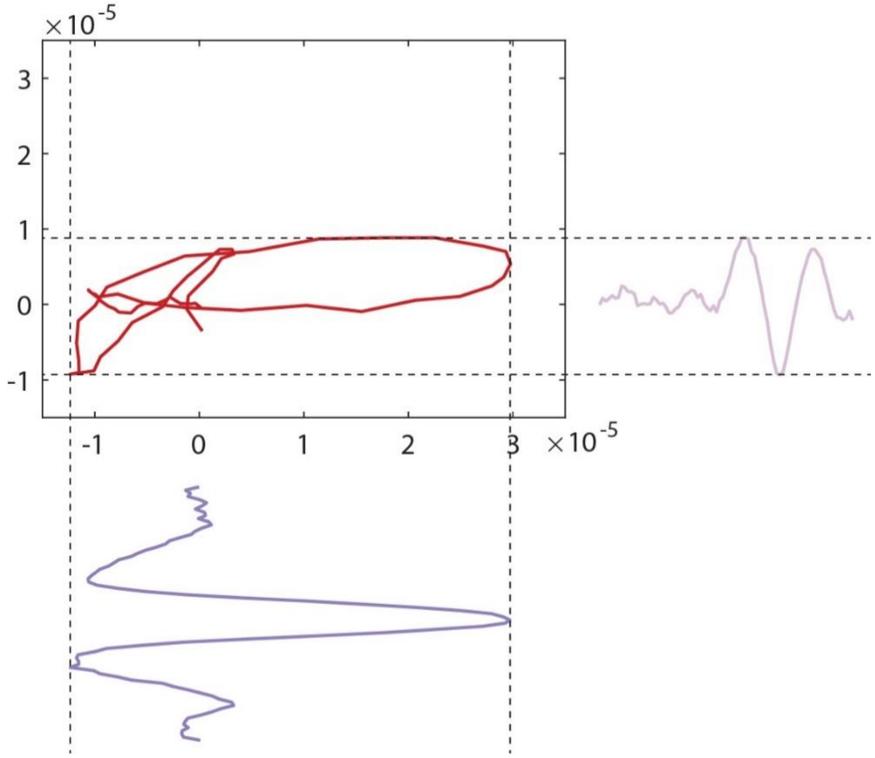

Supplementary Fig. 8 | Lissajous curve of the THz emission pumped with a linearly polarized 800-nm beam.

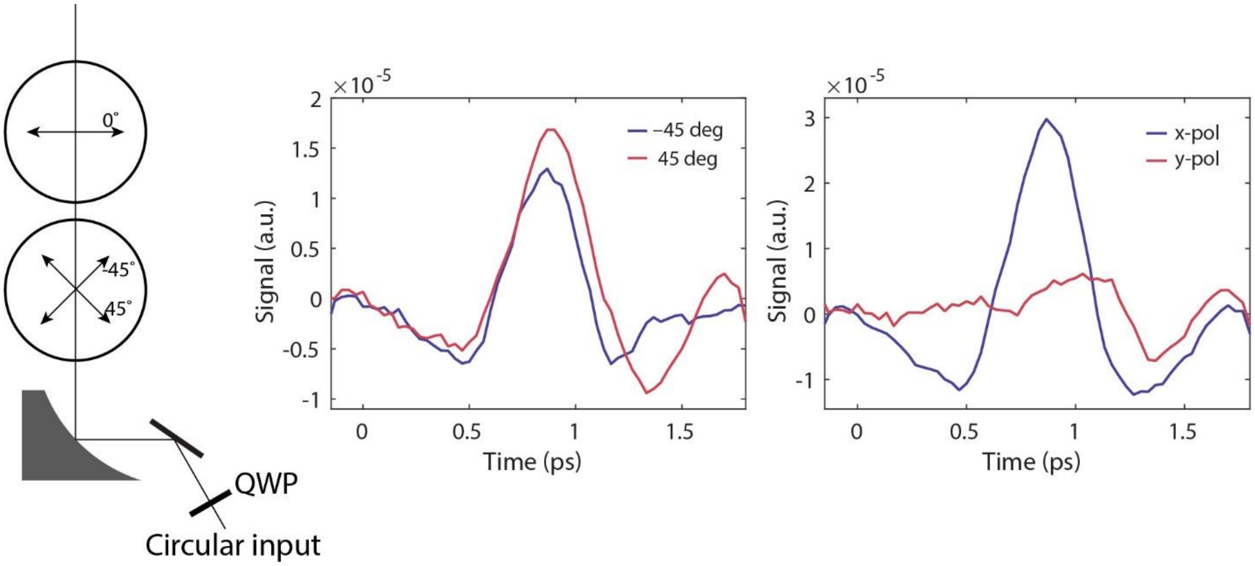

Supplementary Fig. 9 | THz emission polarization analysis with circularly polarized 800-nm pump. **a**, schematic illustration of THz emission polarization analysis with circularly polarized pump. **b**, THz emission traces at 45 degree and -45 degree polarization angles. **c**, THz emission traces with *x* and *y* polarization inferred from **b**, which shows the emission is mainly polarized along the *x*-direction.

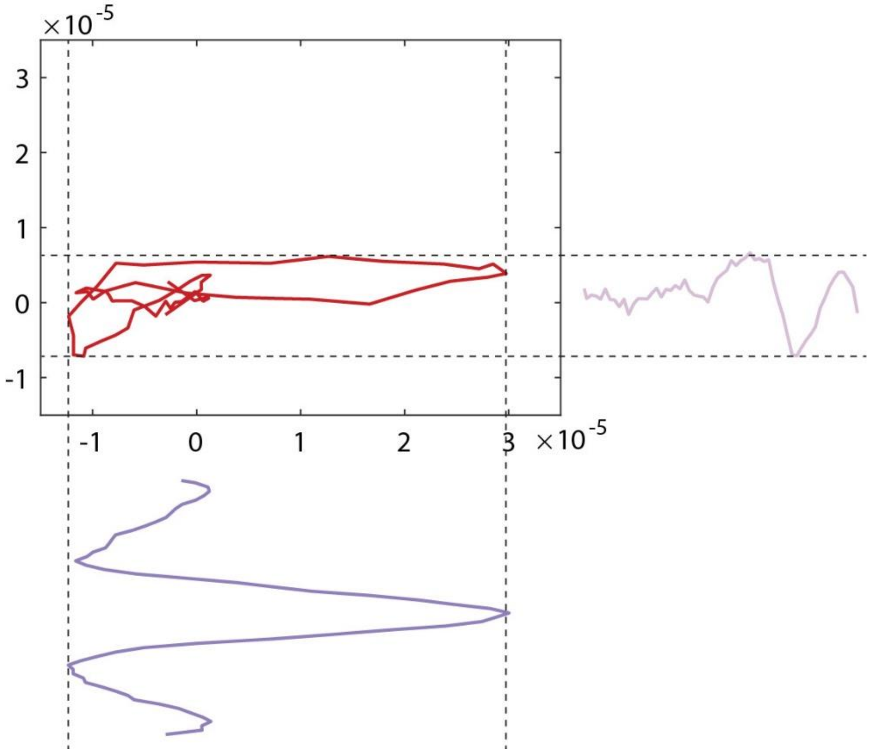

Supplementary Fig. 10 | Lissajous curve of the THz emission pumped with a linearly polarized 800-nm beam. It shows the emission is mainly linearly polarized and along the *x*-direction with a slight ellipticity.

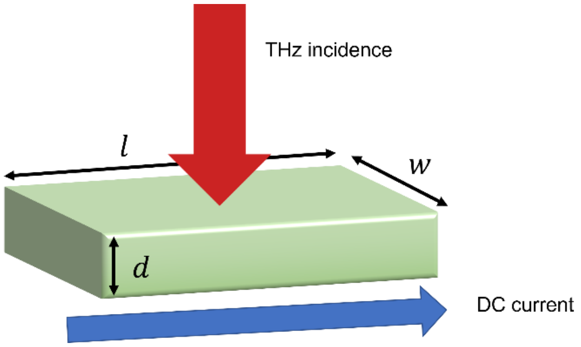

Supplementary Fig. 11 | Sample geometry. l and w are the length and width of the JTMD sample, respectively. d is the thickness of the JTMD monolayer. The THz radiation is along the out-of-plane direction, while the DC current is along the in-plane direction.

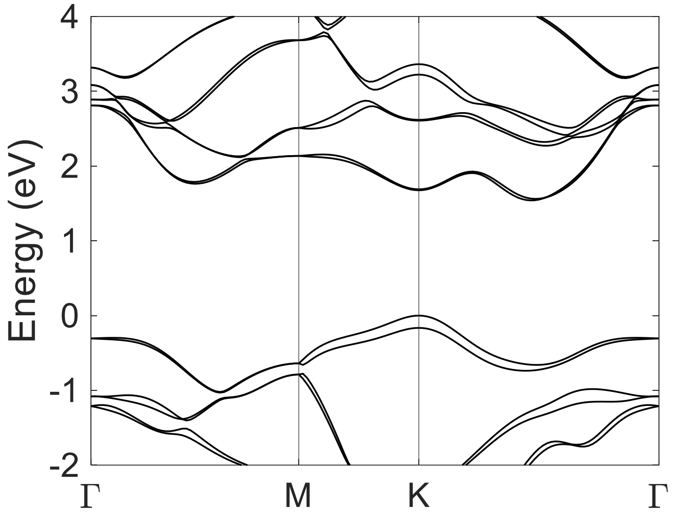

Supplementary Fig. 12 | Calculated electronic band structure of 2*H* MoSSe.

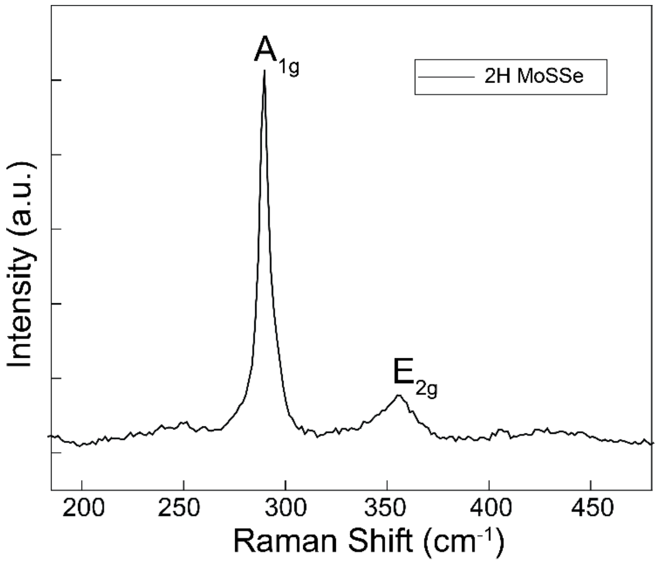

Supplementary Fig. 13 | Raman spectrum of 2*H* MoSSe.

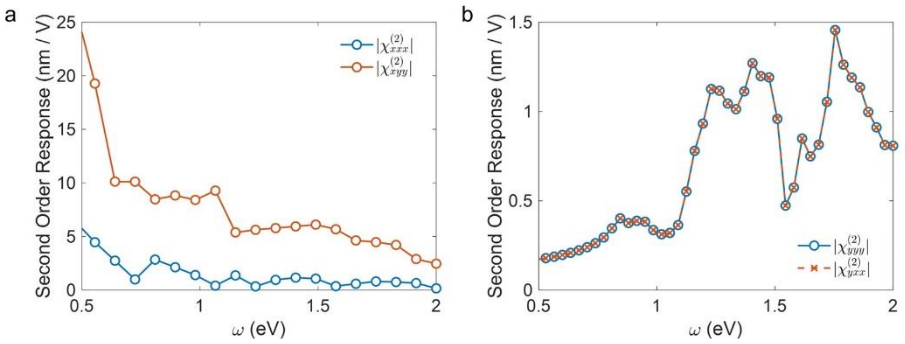

Supplementary Fig. 14 | Non-zero in-plane components of the SHG tensor in (**a**) 1T' and (**b**) 2H MoSSe. Other in-plane components are forbidden by mirror symmetries. In (b), the magnitude of the SHG tensor is the same for $\chi^{(2)}_{yyy}$ and $\chi^{(2)}_{yxx}$ because of the three-fold rotation symmetry in the 2H phase.

Supplementary References

1. Xu, H. *et al.* Colossal switchable photocurrents in topological Janus transition metal dichalcogenides. *npj Comput. Mater.* **7**, 31 (2021).
2. Zhou, J., Xu, H., Li, Y., Jaramillo, R. & Li, J. Opto-Mechanics Driven Fast Martensitic Transition in Two-Dimensional Materials. *Nano Lett.* **18**, 7794–7800 (2018).
3. Zhang, Y. & Fu, L. Terahertz detection based on nonlinear Hall effect without magnetic field. *PNAS* **118**, e2100736118 (2021).
4. Liu, L. *et al.* Phase-selective synthesis of 1*T′* MoS_2 monolayers and heterophase bilayers. *Nat. Mater.* **17**, 1108–1114 (2018).
5. Ghimire, S. *et al.* Observation of high-order harmonic generation in a bulk crystal. *Nat. Phys.* **7**, 138–141 (2011).
6. Ghimire, S. *et al.* Generation and propagation of high-order harmonics in crystals. *Phys. Rev. A* **85**, 043836 (2012).
7. Qian, X., Liu, J., Fu, L. & Li, J. Quantum spin Hall effect in two-dimensional transition metal dichalcogenides. *Science* **346**, 1344–1347 (2014).
8. Ma, E. et al. Recording interfacial currents on the subnanometer length and femtosecond time scale by terahertz emission. *Sci. Adv.* 5, 2 (2019).
